# Quercetin Increases Hepatic Homocysteine Remethylation and Transsulfuration in Rats Fed a Methionine-Enriched Diet

**DOI:** 10.1155/2015/815210

**Published:** 2015-10-19

**Authors:** Bin Meng, Weina Gao, Jingyu Wei, Lingling Pu, Zhenchuang Tang, Changjiang Guo

**Affiliations:** ^1^Tianjin Institute of Health and Environmental Medicine, Tianjin 300050, China; ^2^Tianjin Key Laboratory for Prevention and Control of Occupational and Environmental Hazard, Tianjin 300309, China

## Abstract

This study was aimed at investigating the effects of quercetin on mRNA expression and activity of critical enzymes in homocysteine metabolism in rats fed a methionine-enriched diet. Rats were fed for 6 weeks the following diets, that is, control, 0.5% quercetin, 1.0% methionine, and 1.0% methionine plus 0.5% quercetin diets. Serum homocysteine was significantly increased after methionine treatment and decreased after the addition of quercetin. The mRNA expression of methionine synthase was significantly increased after methionine or methionine plus quercetin supplementation, while its enzymatic activity was significantly increased after methionine plus quercetin supplementation. The mRNA expression and enzymatic activity of cystathionine *β*-synthase and cystathionine *γ*-lyase were upregulated after quercetin, methionine, or quercetin plus methionine treatment and a more significant increase was observed for hepatic cystathionine *β*-synthase in the methionine plus quercetin treated rats, suggesting an interaction between methionine and quercetin. Meanwhile, hepatic ratio of S-adenosylmethionine to S-adenosylhomocysteine was significantly decreased in response to methionine supplementation and normalized after the addition of quercetin. It is concluded that quercetin reduces serum homocysteine by increasing remethylation and transsulfuration of homocysteine in rats exposed to a methionine-enriched diet.

## 1. Introduction

Homocysteine (Hcy) is a nonprotein amino acid, derived from methionine (Met) metabolism [1]. Under the catalysis of methionine adenosyltransferase, Met is converted to S-adenosylmethionine (SAM), which is an important methyl donor* in vivo*. SAM can transfer its methyl group to an acceptor molecule and S-adenosylhomocysteine (SAH) is generated simultaneously. SAH is further hydrolyzed to yield Hcy in a reaction catalyzed by SAH hydrolases. Hcy can be metabolized through two pathways, that is, remethylation and transsulfuration. In remethylation pathway, Hcy can be remethylated to form Met via methionine synthase (MS) or betaine-homocysteine methyltransferase (BHMT), in which cofactors such as folic acid and vitamin B_12_ or betaine are required. In transsulfuration pathway, Hcy is reacted with serine (Ser) to form cystathionine via vitamin B_6_-dependent cystathionine *β*-synthase (CBS). Subsequently, cystathionine is hydrolyzed to yield cysteine (Cys) and *α*-ketobutyrate via vitamin B_6_-dependent cystathionine *γ*-lyase (CSE) and finally to taurine (Tau) [2, 3].

Hyperhomocysteinemia (HHcy) is associated with genetic and nutritional abnormalities in Hcy metabolism and has been demonstrated to be an independent risk factor for cardiovascular disease and several other diseases [4–7]. Among the potential mechanisms proposed, the role of reactive oxygen species released during the autooxidation of the thiol group of Hcy has been frequently mentioned in HHcy related diseases [8, 9]. Recently, DNA hypomethylation also has been hypothesized to be associated with the actions Hcy plays* in vivo*, because SAH, a potent inhibitor of cellular methylation, was found to be increased in HHcy [10]. However, it was reported that Hcy-lowering interventions in the form of supplements of folic acid or vitamin B_6_ or B_12_ given alone or in combination were not effective in the prevention against cardiovascular events, such as myocardial infarction and stroke or death by any cause [11]. Moreover, Smulders and Blom suggested that high dose of B vitamins might be harmful to vascular endothelial cells. In particular, folic acid, a synthetic provitamin, may have adverse effects at high intake levels on normal transmembrane folate transport, inflammatory and proliferating cells in atherosclerotic lesions [12]. Therefore, it is important to find new alternatives in the prevention or treatment of HHcy.

Quercetin, a common member of the flavonoids family, is distributed ubiquitously in plant kingdom and rich especially in apples, teas, and onions. Several epidemiological studies showed that quercetin intake was inversely associated with the risk of cardiovascular disease, which has been contributed possibly by its antioxidant and anti-inflammatory properties, inhibition of LDL oxidation, and platelet aggregation [13–15]. Previously, we demonstrated that quercetin was effective in reducing serum Hcy level in rats fed a Met-enriched diet, which may also contribute to its protection against cardiovascular disease [16]. However, the underlying mechanisms were not yet fully examined. The objective of the present study was to further investigate the effects of quercetin on mRNA expression and activity of critical enzymes in Hcy metabolism. Meanwhile, the changes of relevant metabolites in Met metabolism were also measured so as to validate the action of quercetin. Our results demonstrated that quercetin enhanced hepatic Hcy remethylation and transsulfuration in rats exposed to a Met-enriched diet.

## 2. Materials and Methods

### 2.1. Chemicals

Adenosine deaminase, 4-chloro-7-sulfobenzofurazan ammonium salt (SBD-F), L-Cys, cystathionine, Hcy, heptanesulfonate, hydroxocobalamin, L-Met, 5-methyltetrahydrofolate, propargylglycine, pyridoxal 5′-phosphate, quercetin, reduced glutathione (GSH), SAH, SAM, and L-Ser were obtained from Sigma-Aldrich, Inc. (St. Louis, MO, USA). TRIzol reagent kit and PCR primers for MS, BHMT, CBS, and CSE were purchased from Takara (Dalian, China). FastStart Universal SYBR Green Master mix and cDNA synthesis kit were obtained from Roche (Roche, Switzerland). All other chemicals were of the highest grade available.

### 2.2. Animals, Diets, and Experimental Protocol

Animal handling was performed according to the current Chinese legislation on the care and use of laboratory animals. The experimental protocol was approved by the Ethical Committee of the Department of Scientific Management of the institute. Thirty-two male Wistar rats, weighing 180 g–205 g, were purchased from the Laboratory Animal Center, Chinese Academy of Military Medical Sciences (Beijing, China), and housed individually in stainless-steel cages. The room temperature was controlled between 18°C and 24°C and relative humidity between 40% and 60%. The light/dark cycles were alternated every 12 h. Food and tap water were provided* ad libitum*. Dietary intake and body weight were recorded every day. After being acclimatized on a polyphenol-free semisynthetic diet (AIN-93 formula) [17] for 5 days, the rats were divided randomly into four groups based on fasting serum Hcy levels and maintained for 6 weeks on the following diets: control diet (AIN-93 diet), 0.5% quercetin supplemented AIN-93 diet (0.5% Q), 1.0% Met supplemented AIN-93 diet (1.0% Met), and 1.0% Met plus 0.5% quercetin supplemented AIN-93 diet (1.0% Met/0.5% Q). The loading dose of 1.0% Met we chose to induce HHcy was based on the studies conducted by Velez-Carrasco et al. and Toue et al. [18, 19]. In the previous study, 0.5% quercetin had been proved to be effective in reducing plasma Hcy in rats fed a Met-enriched AIN-93 diet [16]. At the end of the experiment, all rats were fasted overnight and blood samples were collected from the orbital plexus under ether anesthetization. The serum was separated and stored at −20°C. Rat livers were also sampled immediately, cleaned up in ice cold saline, and snap-frozen at −80°C prior to analysis.

### 2.3. Serum Contents of Met, Hcy, Ser, Tau, Gly, Cys, and GSH

Serum Met, Ser, Tau, and Gly were measured using an automatic amino acid analyzer (Hitachi L-8800, Tokyo, Japan) based on cation-exchange chromatography and post-column reaction with ninhydrin reagent. Serum Hcy, Cys, and GSH were analyzed by a HPLC method reported by Krijt et al. [20].

### 2.4. Hepatic Contents of SAH and SAM

Hepatic SAH and SAM were measured by a HPLC procedure described by She et al. [21]. Ultraviolet absorbance was measured at 245 nm. The SAH and SAM peaks were identified and quantified.

### 2.5. Hepatic Activities of MS, BHMT, CBS, and CSE

Hepatic MS and BHMT activities were assayed according to the methods developed by Drummond et al. [22] and Yagisawa et al. [23], respectively. Hepatic CBS and CSE activities were determined based on the procedures reported by Zou and Banerjee [24] and Bravo et al. [25], respectively.

### 2.6. Hepatic mRNA Expressions of MS, BHMT, CBS, and CSE

Total RNA was extracted by using the TRIzol reagent. The first strand cDNA was synthesized by using cDNA synthesis kit. Quantitative real-time PCR was performed after cDNA synthesis in 25 *μ*L of a FastStart Universal SYBR Green Master mix. The primers used for the genes studied are shown in Table 1. The thermal cycling conditions used were as follows: an initial DNA denaturation step at 95°C for 5 seconds, followed by 40 cycles of denaturation at 95°C for 5 seconds, primer annealing at optimal temperature for 20 s, extension at 72°C for 30 s, and an additional incubation step at 80–85°C for 30 s to measure SYBR Green I fluorescence. Finally, melting curve analysis was performed by slowly cooling the PCR from 95°C to 60°C (0.5°C per cycle) with simultaneous measurement of the SYBR Green I signal intensity. The ΔCt method was used to evaluate the relative quantification. The value for each sample was determined by calculating the difference between the Ct value of the target gene and the Ct value of the *β*-actin reference gene. The normalized target gene expression level in the sample was calculated by using the formula 2^−ΔΔCt(2ΔCt  (actin)−ΔCt  (target  gene))^.

### 2.7. Statistical Analysis

All data are expressed as mean and standard deviation. The statistical analysis was performed using the SPSS 10.01 software (SPSS Inc., Chicago, IL, USA). Two-way ANOVA was carried out with* post hoc* Bonferroni *t*-test. Differences between treatments were considered to be statistically significant at *p* < 0.05.

## 3. Results

### 3.1. Dietary Intake and Body Weight

No significant difference was found in dietary intake and body weight among the four groups during the experimental period, indicating that 1.0% Met or/and 0.5% quercetin supplementations do not affect significantly rat food consumption or growth (Table 2).

### 3.2. Serum Met, Hcy, Ser, Cys, Gly, Tau, and GSH

As shown in Table 3, serum Met was not changed significantly in exposure either to 0.5% quercetin, to 1.0% Met, or to 1.0% Met plus 0.5% quercetin treatment. Serum Hcy was increased significantly after 1.0% Met supplementation. In comparison to 1.0% Met supplemented rats, serum Hcy was reduced by 27.6% in 1.0% Met plus 0.5% quercetin treated rats (*p* < 0.05), which is consistent with the data we reported previously [16]. Serum Ser and Gly were decreased significantly by 30.3% and 17.8%, respectively, after 1.0% Met plus 0.5% quercetin supplementation (*p* < 0.05). As compared with the control group, serum content of Cys was increased significantly by 91.9%, 125.6%, and 124.9%, respectively, in 0.5% quercetin, 1.0% Met, and 1.0% Met plus 0.5% quercetin (*p* < 0.05), while serum content of Tau was increased significantly by 20.2%, 31.8%, and 46.6%, respectively (*p* < 0.05). Serum GSH was significantly declined by 20.7% after 0.5% quercetin treatment and increased by 18.8% after 1.0% Met supplementation. A 29.6% decrease in serum GSH was found after 1.0% Met plus 0.5% quercetin treatment compared to 1.0% Met treatment.

### 3.3. Hepatic SAM, SAH and Ratio of SAM to SAH

Hepatic SAM was significantly increased by 41.8% after 1.0% Met plus 0.5% quercetin supplementation (*p* < 0.05), whereas hepatic SAH was significantly increased by 34.5% and 29.4%, respectively, after 1.0% Met and 1.0% Met plus 0.5% quercetin supplementation (*p* < 0.05). The ratio of SAM to SAH was decreased after 1.0% Met supplementation and recovered upon the addition of 0.5% quercetin (Table 4).

### 3.4. Hepatic MS mRNA Expression and Activity

**Figure 1:**
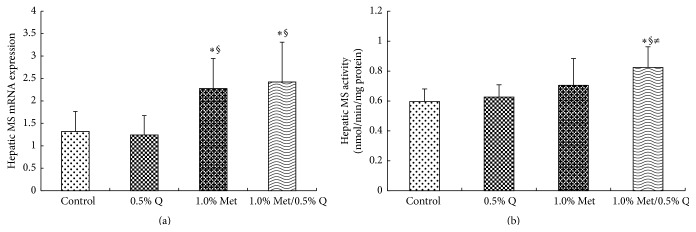
Hepatic MS mRNA expression and activity. MS, methionine synthase; 0.5% Q, 0.5% quercetin supplemented group; 1.0% Met, 1.0% Met supplemented group; 1.0% Met/0.5% Q, 1.0% Met plus 0.5% quercetin supplemented group. (a) Effect of quercetin on hepatic MS mRNA expression in rats fed a Met-enriched diet for 6 weeks. The mRNA expression of MS was normalized to *β*-actin. (b) Effect of quercetin on hepatic MS activity in rats fed a Met-enriched diet for 6 weeks. Results are expressed as mean and standard deviation (*n* = 8). ^*∗*^Significantly different from the control (*p* < 0.05). ^§^Significantly different from the 0.5% Q group (*p* < 0.05). ^≠^Significantly different from the 1.0% Met group (*p* < 0.05).

Hepatic mRNA expression of MS was significantly higher by 72.7% and 83.3% in 1.0% Met and 1.0% Met plus 0.5% quercetin supplemented rats than in the control (*p* < 0.05). Hepatic MS activity was significantly increased by 39.0% in 1.0% Met plus 0.5% quercetin supplemented rats compared to those in the control group (*p* < 0.05), suggesting an interaction between quercetin and Met. No significant change was found for hepatic MS activity in 1.0% Met or 0.5% quercetin supplemented rats when compared to the control rats (Figure 1).

### 3.5. Hepatic BHMT mRNA Expression and Activity

**Figure 2:**
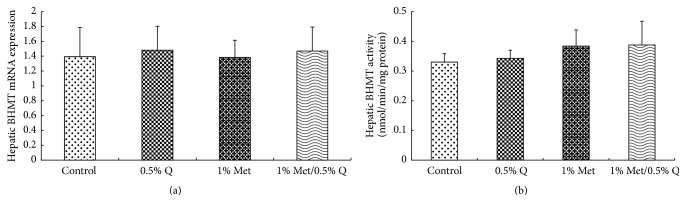
Hepatic BHMT mRNA expression and activity. BHMT, betaine-homocysteine methyltransferase; 0.5% Q, 0.5% quercetin supplemented group; 1.0% Met, 1.0% Met supplemented group; 1.0% Met/0.5% Q, 1.0% Met plus 0.5% quercetin supplemented group. (a) Effect of quercetin on hepatic BHMT mRNA expression in rats fed a Met-enriched diet for 6 weeks. The mRNA expression of BHMT was normalized to *β*-actin. (b) Effect of quercetin on hepatic BHMT activity in rats fed a Met-enriched diet for 6 weeks. Results are expressed as mean and standard deviation (*n* = 8). No significant difference was found among the four groups.

As indicated in Figure 2, no significant difference was showed in hepatic BHMT mRNA expression and activity among different groups, demonstrating that 1.0% Met or/and 0.5% quercetin supplementations do not affect hepatic BHMT mRNA expression and activity.

### 3.6. Hepatic mRNA Expression and Activity of CBS and CSE

**Figure 3:**
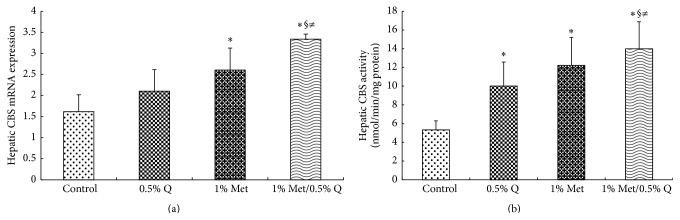
Hepatic CBS mRNA expression and activity. CBS, cystathionine *β*-synthase; 0.5% Q, 0.5% quercetin supplemented group; 1.0% Met, 1.0% Met supplemented group; 1.0% Met/0.5% Q, 1.0% Met plus 0.5% quercetin supplemented group. (a) Effect of quercetin on hepatic CBS mRNA expression in rats fed a Met-enriched diet for 6 weeks. The mRNA expression of CBS was normalized to *β*-actin. (b) Effect of quercetin on hepatic CBS activity in rats fed a Met-enriched diet for 6 weeks. Results are expressed as mean and standard deviation (*n* = 8). ^*∗*^Significantly different from the control (*p* < 0.05). ^§^Significantly different from the 0.5% Q group (*p* < 0.05). ^≠^Significantly different from the 1% Met group (*p* < 0.05).

**Figure 4:**
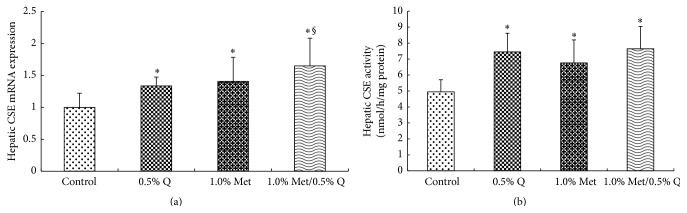
Hepatic CSE mRNA expression and activity. CSE, cystathionine *γ*-lyase; 0.5% Q, 0.5% quercetin supplemented group; 1.0% Met, 1.0% Met supplemented group; 1.0% Met/0.5% Q, 1.0% Met plus 0.5% quercetin supplemented group. (a) Effect of quercetin on hepatic CSE mRNA expression in rats fed a Met-enriched diet for 6 weeks. The mRNA expression of CSE was normalized to *β*-actin. (b) Effect of quercetin on hepatic CSE activity in rats fed a Met-enriched diet for 6 weeks. Results are expressed as mean and standard deviation (*n* = 8). ^*∗*^Significantly different from the control (*p* < 0.05). ^§^Significantly different from the 0.5% Q group (*p* < 0.05).

Hepatic mRNA expression of CBS was significantly enhanced by 60.5% or 106.2% after 1.0% Met or 1.0% Met plus 0.5% quercetin supplementation compared to the control group (*p* < 0.05). Comparatively, more significant increase in CBS mRNA expression was noted in 1.0% Met plus 0.5% quercetin supplemented rats (Figure 3). On the other hand, hepatic mRNA expression of CSE was increased significantly by 34.3%, 42.4%, and 66.7%, respectively, after 0.5% quercetin, 1.0% Met, and 1.0% Met plus 0.5% quercetin supplementations (*p* < 0.05) and a more significant increase was seen in 1.0% Met plus 0.5% quercetin supplemented rats compared to 0.5% quercetin supplemented rats (Figure 4) (*p* < 0.05). Both hepatic CBS and CSE activities were significantly increased after 0.5% quercetin, 1.0% Met, and 1.0% Met plus 0.5% quercetin supplementations (*p* < 0.05) and a more significant increase in hepatic CBS activity was found in 1.0% Met plus 0.5% quercetin supplementation than in 0.5% quercetin or 1.0% Met supplementation (162.3% versus 87.8% or 129.1%) (*p* < 0.05), indicating an interaction between quercetin and Met (Figures 3 and 4).

## 4. Discussion

Previously, we found that the diet supplemented with 0.5% quercetin was effective in reducing serum Hcy level in rats exposed to a Met-enriched diet, indicating that quercetin has the potential in the prevention or treatment against HHcy [16]. The present study, for the first time, reported that quercetin supplementation was effective in modulating hepatic mRNA expression and activity of several critical enzymes in Hcy metabolism.

After Met supplementation, it was showed that serum Hcy and hepatic SAH were increased significantly, while an increasing trend was noted for SAM. This is consistent with the data reported previously by others [26], confirming that excess Met induces a significant disturbance in Hcy metabolism. After quercetin treatment, both SAM and SAH did not change significantly in the liver, though SAM showed an increasing trend. It is not surprising because the gastrointestinal tissues, not the liver, are the main place where quercetin is glucuronidated, sulfated, and methylated extensively upon absorption [27]. The portal blood contained only quercetin metabolites after quercetin administration in pigs, suggesting that quercetin is biotransformed in gastrointestinal tissues before reaching the liver [28]. Some of the quercetin metabolites may retain the ability in stimulating the synthesis of SAM after entering the liver, because only the liver is responsible in producing more SAM endogenously [2, 3]. After Met plus quercetin treatment, both SAM and SAH were increased significantly in the liver, which resulted from the combined action of Met and quercetin metabolites. It had been demonstrated that the ratio of SAM to SAH is crucial in the regulation of multiple enzymatic transmethylation reactions [29]. A decrease of SAM/SAH ratio is associated with inhibition of transmethylation reactions, which will affect the biosynthesis of some proteins, hormones, phospholipids, neurotransmitters, RNA, and DNA [30–32]. In the present study, it was noted that Met supplementation decreased the SAM/SAH ratio, which was normalized after Met plus quercetin supplementation. Thereby, quercetin was beneficial to the liver by upregulating transmethylation reactions in rats exposed to excess Met.

Both SAM and SAH play an important role in Hcy remethylation. SAM is an allosteric inhibitor of methylenetetrahydrofolate reductase (MTHFR) and suppresses the synthesis of 5-methyltetrahydrofolate, an important substrate required for the remethylation of Hcy. Thereby, SAM is generally considered an inhibitor for MS [1–3, 33]. In this study, a significant increase in MS mRNA expression was found in the liver after Met supplementation, though no significant increase was noted for MS activity. This seems not consistent with data reported previously by others, in which hepatic MS was inhibited after exposure to excess Met [2, 33]. It is explainable because hepatic SAM was not increased significantly after Met supplementation in the current study. Meanwhile, a simultaneous increase was noted for hepatic SAH, which is an activator for MTHFR [33]. Moreover, it had been demonstrated* in vitro* that SAM was required to be a cofactor for MS and stimulated the formation of product by MS [26, 33, 34]. A similar result had been reported by Di Buono et al., in which they demonstrated that Hcy remethylation was promoted in response to increasing Met intake in men [35]. After quercetin treatment, no remarkable change was noted in hepatic MS mRNA expression and activity. However, the combination of Met and quercetin treatments could increase significantly both hepatic MS mRNA expression and activity, though SAM was also increased significantly. A simultaneous increase in hepatic SAH is partially responsible, as explained above. Additionally, a significant decrease in serum Gly was found after Met plus quercetin treatment in this study, indicating that more glycine was transformed via Gly* N*-methyltransferase (GNMT), a most abundant methyltransferase in the liver [36]. Wang et al. demonstrated that increased GNMT activity could improve folate retention and bioavailability in the liver and resulted in enhanced Hcy remethylation [37, 38]. We are regretful that the GNMT activity was not measured in this study and further experiments are needed to investigate the effects of quercetin on GNMT.

CBS, a vitamin B_6_-dependent enzyme, catalyzes the condensation of Hcy with Ser to form cystathionine in the transsulfuration pathway of Hcy metabolism. It was found that both SAM and SAH could function as an allosteric activator of CBS [1–3]. Moreover, two sets of SAM-binding sites in the C-terminal regulatory domain were found recently in CBS, in which a high affinity set was responsible for kinetic stabilization of the domain and a low affinity set was involved in the enzyme activation. Therefore, SAM binding leads to both CBS stabilization and activation [39]. In the present study, we noted a significant increase in both mRNA expression and activity of hepatic CBS in the Met treated rats and a more significant increase was observed in the Met plus quercetin treated rats, suggesting an interaction between Met and quercetin. One of possible mechanisms was associated with significantly increased hepatic SAM after Met plus quercetin treatment. Meanwhile, serum Ser was decreased significantly, which is associated with increased activity of CBS.

CSE, another vitamin B_6_-dependent enzyme in the transsulfuration pathway, catalyzes the conversion of cystathionine into Cys and *α*-ketobutyrate [2]. We found that both CSE mRNA expression and activity were significantly increased in response to 0.5% quercetin, 1.0% Met, or 1.0% Met plus 0.5% quercetin treatment. In the meantime, serum Cys and Tau were significantly increased, which is in line with increased CSE activity and indicates that Hcy transsulfuration is enhanced after exposure to quercetin or/and Met treatments in rats. However, no clear interaction between quercetin and Met was demonstrated on CSE mRNA expression and activity.

GSH, an important antioxidant, is synthesized from three amino acids, Cys, Gly, and glutamic acid. It was reported that quercetin could protect GSH against oxidation [40]. However, quercetin at high intake levels increased GSH degradation since quercetin could be oxidized to form a thiol-reactive quinone and led to increased GSH consumption preferentially [41, 42]. We also found that the content of GSH in serum or liver tissue was decreased in response to quercetin administration in rats fed a Met-enriched diet [16]. Supporting results were obtained again from the present study, in which serum GSH was significantly declined after quercetin administration. It is suggested that the redox state is altered by quercetin treatment. It is of interest because the CBS enzyme contains a heme cofactor in the N-terminal domain that functions as a redox sensor [43]. Quercetin treatment may alter the redox state of the heme and thereby activate CBS indirectly.

**Figure 5:**
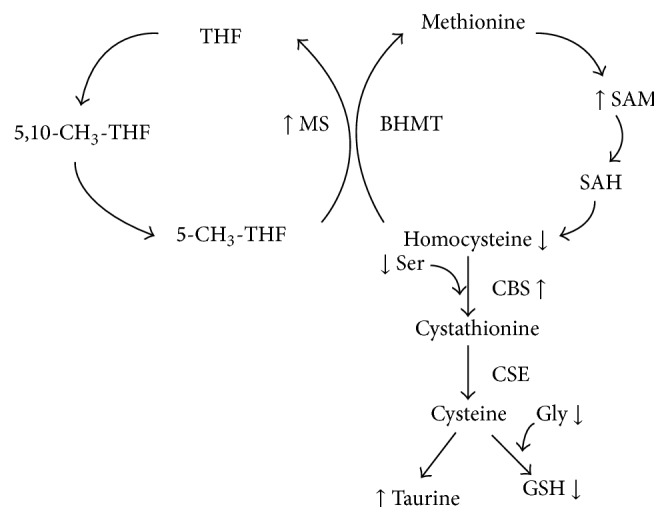
Possible targets of quercetin in homocysteine metabolic pathways in rats fed a Met-enriched diet. MS, methionine synthase; BHMT, betaine-homocysteine methyltransferase; CBS, cystathionine *β*-synthase; CSE, cystathionine *γ*-lyase; SAH, S-adenosylhomocysteine; SAM, S-adenosylmethionine; Cys, cysteine; Tau, taurine; Ser, serine; Gly, glycine; GSH, reduced glutathione; THF, tetrahydrofolate; 5,10-CH_3_-THF, 5,10-CH_3_-tetrahydrofolate; 5-CH_3_-THF, 5-CH_3_-tetrahydrofolate. The arrows (↑ or ↓) indicate the changes (increase or decrease) after quercetin treatment.

In conclusion, this study demonstrates that quercetin can enhance Hcy remethylation and transsulfuration in rats fed a Met-enriched diet by increasing hepatic mRNA expression and activity of MS and CBS, two critical enzymes in Hcy metabolism (Figure 5). The data generated in the current study would be applicable to humans because it has been demonstrated that high protein or Met diet could increase postprandial plasma total Hcy concentrations in humans [44]. More studies are needed to investigate the molecular mechanisms whereby quercetin regulates the mRNA expression and activity of MS and CBS. It is also worthwhile to further validate the effects of quercetin on HHcy in other animal models, including those induced by the insufficiency of B vitamins, such as folate, vitamin B_6_, or vitamin B_12_ alone or in combination.

## Figures and Tables

**Table 1:** The primers used in real-time PCR.

Gene	GenBank		Primers
MS	NM_030864.1	F	5′-GGATCTCTGGGTCCGACTAACAA-3′
		R	5′-GCCTGCTCCTGGTATGCTTCA-3′

BHMT	NM_030850.1	F	5′-GAACCAGAGTTGCCACCAGATG-3′
		R	5′-AGCAGCCGCCAATGTACCTGAC-3′

CBS	NM_012522.2	F	5′-AAGGCTGCCCAGGAGCTAAG-3′
		R	5′-CAGCATCCATTTGTCACTCAAGAAC-3′

CSE	NM_017074.1	F	5′-GTCCACAAACACAAAGACATCA-3′
		R	5′-AGGTCATCGGAAGTAACAGACA-3′

*β*-actin	NM_007393.1	F	5′-GTCCCTCACCCTCCCAAAA-3′
		R	5′-GCTGCCTCAACACCTCAACCC-3′

MS, methionine synthase; BHMT, betaine-homocysteine methyltransferase; CBS, cystathionine *β*-synthase; CSE, cystathionine *γ*-lyase; F, forward; R, reverse.

**Table 2:** Changes of body weight and dietary intake during experimental period.

Group	Body weight (g)		Dietary intake (g/d)
	Initial	Final	
Control	191.1 ± 6.7	362.9 ± 8.4	17.4 ± 4.4
0.5% Q	191.5 ± 7.5	362.8 ± 6.8	17.7 ± 2.9
1.0% Met	194.8 ± 7.6	364.3 ± 8.2	17.5 ± 1.6
1.0% Met/0.5% Q	194.4 ± 7.7	363.7 ± 6.4	17.3 ± 2.8

0.5% Q, 0.5% quercetin supplemented group; 1.0% Met, 1.0% Met supplemented group; 1.0% Met/0.5% Q, 1.0% Met plus 0.5% quercetin supplemented group.

Results are expressed as mean and standard deviation (*n* = 8). No significant difference was found among the four groups.

**Table 3:** Serum contents of Met, Hcy, Cys, Tau, Ser, Gly, and GSH.

Group	Met (*μ*M/L)	Hcy (*μ*M/L)	Cys (*μ*M/L)	Tau (*μ*M/L)	Ser (*μ*M/L)	Gly (*μ*M/L)	GSH (*μ*M/L)
Control	75.7 ± 4.7	3.4 ± 0.6	29.7 ± 6.7	344.4 ± 37.6	316.9 ± 42.8	426.3 ± 79.9	25.6 ± 5.7
0.5% Q	68.4 ± 2.0	3.7 ± 0.4	57.0 ± 14.2^*∗*^	413.9 ± 15.2^*∗*^	287.4 ± 64.7	385.0 ± 89.3	20.3 ± 3.1^*∗*^
1.0% Met	68.4 ± 5.4	5.8 ± 1.1^*∗*§^	67.0 ± 9.0^*∗*§^	454.0 ± 46.3^*∗*^	285.5 ± 27.6	419.6 ± 63.9	30.4 ± 3.3^*∗*§^
1.0% Met/0.5% Q	69.0 ± 0.7	4.2 ± 0.7^≠^	66.8 ± 3.9^*∗*§^	505.0 ± 31.2^*∗*§≠^	220.8 ± 22.8^*∗*§≠^	350.3 ± 66.6^*∗*≠^	21.4 ± 2.5^≠^

Met, methionine; Hcy, homocysteine; Cys, cysteine; Tau, taurine; Ser, serine; Gly, glycine; GSH, reduced glutathione; 0.5% Q, 0.5% quercetin supplemented group; 1.0% Met, 1.0% Met supplemented group; 1.0% Met/0.5% Q, 1.0% Met plus 0.5% quercetin supplemented group.

Results are expressed as mean and standard deviation (*n* = 8). ^*∗*^Significantly different from the control group (*p* < 0.05). ^§^Significantly different from the 0.5% Q group (*p* < 0.05). ^≠^Significantly different from the 1.0% Met group (*p* < 0.05).

**Table 4:** Hepatic contents of SAM, SAH and the ratio of SAM to SAH.

Group	SAM (nmol/g liver)	SAH (nmol/g liver)	SAM/SAH
Control	106.9 ± 23.1	82.9 ± 7.8	1.3 ± 0.2
0.5% Q	129.2 ± 19.8	81.9 ± 10.3	1.6 ± 0.5
1% Met	125.3 ± 15.3	111.5 ± 9.3^*∗*§^	1.1 ± 0.2^§^
1% Met/0.5% Q	151.6 ± 13.4^*∗*≠^	107.3 ± 9.2^*∗*§^	1.4 ± 0.2

SAH, S-adenosylhomocysteine; SAM, S-adenosylmethionine; 0.5% Q, 0.5% quercetin supplemented group; 1.0% Met, 1.0% Met supplemented group; 1.0% Met/0.5% Q, 1.0% Met plus 0.5% quercetin supplemented group. Results are expressed as mean and standard deviation (*n* = 8). ^*∗*^Significantly different from the control group (*p* < 0.05). ^§^Significantly different from the 0.5% Q group (*p* < 0.05). ^≠^Significantly different from the 1.0% Met group (*p* < 0.05).
